# Temozolomide is additive with cytotoxic effect of irradiation in canine glioma cell lines

**DOI:** 10.1002/vms3.620

**Published:** 2021-09-03

**Authors:** Nina Simona Tresch, Daniel Fuchs, Luca Morandi, Caterina Tonon, Carla Rohrer Bley, Katarzyna J. Nytko

**Affiliations:** ^1^ Division of Radiation Oncology Vetsuisse Faculty University of Zurich Zurich Switzerland; ^2^ Center for Clinical Studies at the Vetsuisse Faculty of the University of Zurich Zurich Switzerland; ^3^ Department of Biomedical and Neuromotor Sciences University of Bologna Bologna Italy; ^4^ Functional and Molecular Neuroimaging Unit IRCCS Istituto delle Scienze Neurologiche di Bologna Bologna Italy

**Keywords:** brain tumour, chemoradiation, dog, in vitro, O‐6‐methylguanine‐DNA methyltransferase (MGMT)

## Abstract

**Background:**

Similar to human glioblastoma patients, glial tumours in dogs have high treatment resistance and a guarded prognosis. In human medicine, the addition of temozolomide to radiotherapy leads to a favourable outcome in vivo as well as a higher antiproliferative effect on tumour cells in vitro.

**Objectives:**

The aim of the study was to determine the radio‐ and temozolomide‐sensitivity of three canine glial tumour cell lines and to investigate a potential additive cytotoxic effect in combined treatment. Additionally, we wanted to detect the level of *MGMT* promoter methylation in these cell lines and to investigate a potential association between *MGMT* promoter methylation and treatment resistance.

**Methods:**

Cells were treated with various concentrations of temozolomide and/or irradiated with 4 and 8 Gy. Radiosensitization by temozolomide was evaluated using proliferation assay and clonogenic assay, and *MGMT* DNA methylation was investigated using bisulfite next‐generation sequencing.

**Results:**

In all tested canine cell lines, clonogenicity was inhibited significantly in combined treatment compared to radiation alone. All canine glial cell lines tested in this study were found to have high methylation levels of *MGMT* promoter.

**Conclusions:**

Hence, an additive effect of combined treatment in MGMT negative canine glial tumour cell lines in vitro was detected. This motivates to further investigate the association between treatment resistance and MGMT, such as *MGMT* promoter methylation status.

## Graphical abstract

Temozolomide pre‐treatment enhanced the cytotoxic effect or irradiation in canine glioma cell lines. Clonogenic cell survival assay was used as a readout.

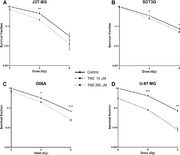

## 1 INTRODUCTION

Similar to human glioblastoma, canine glial tumors are associated with high treatment resistance and guarded overall outcome. Single‐modality radiation therapy or surgery often represents the most promising strategy with median survival times of 8–23 months (Debreuque et al., 2020; Hubbard et al., 2018; Schwarz et al., 2018). Other than in human medicine and rodent models using human xenografts, possible benefit of combined treatment has not been described. Chemotherapeutic agents are only sporadically used, without ameliorating outcome and without consensus on drug type or dose (Hu et al., 2015).

In human glioblastoma patients, the chemotherapeutic agent temozolomide (TMZ) improves outcome when added to tumour resection and radiation therapy (Stupp et al., 2009). Temozolomide as an alkylating agent methylates the DNA most often at the N^7^ or O^6^ positions of guanine residues and at the O^3^ site on adenine. This leads to DNA damage and triggers tumour cell death (Clark et al., 1995; Knizhnik et al., 2013; Wang et al., 2016; Zhou et al., 2007). However, some tumour cells are able to repair this type of DNA damage using a repair protein O^6^‐alkylguanine DNA alkyltransferase, which is encoded by the *O‐6‐methylguanine‐DNA methyltransferase* (*MGMT*) gene (Kaina & Christmann, 2002; Lee, 2016; Perazzoli et al., 2015). Epigenetic silencing (methylation) of the *MGMT* promoter results in decreased synthesis of MGMT and in turn lowers the DNA‐repair capacity of the cell. Treatment‐induced DNA damage in tumour cells with low MGMT levels can therefore no longer be repaired. Hence, in glioblastoma patients with methylated *MGMT* promoter, combined (chemoradiation) treatment is more efficient, and a survival advantage can be observed compared to patients with unmethylated *MGMT* promoter (Chinot et al., 2007; Eoli et al., 2007; Hegi et al., 2005). In canine glioma, both local recurrences, as well as CNS‐metastasis after radiation therapy has been observed clinically (Dolera et al., 2018; Rohrer Bley et al., 2021; Schwarz et al., 2018). Neither in vivo nor in vitro, however, the presence and possible importance of the *MGMT* gene in dogs' glioma treatment response or progression pattern have been investigated up to date. Hence, we do not know, if silencing a *MGMT* promoter could lead to the same survival advantage as described in human patients treated with radiotherapy and/or alkylating agent.

The purpose of this in vitro cell culture study is to test the response of canine glial tumour cells towards temozolomide, radiotherapy and combination of both, as well as to investigate the potential involvement of MGMT in the mechanism of sensitization.

## 2 MATERIALS AND METHODS

### 2.1 Cell line validation statement and culture conditions

Human glial tumour cell lines A172 and U‐87 MG were obtained from The European Collection of Authenticated Cell Cultures (ECACC, Sigma‐Aldrich). Canine glial tumour cell lines J3T‐BG, SDT3G and G06A were a kind gift of PD Philippe Plattet, PhD (Division of Neurological Sciences, Vetsuisse Faculty, University of Bern, Bern, Switzerland) and were previously described (York et al., 2012). A172 cells were maintained in DMEM cell culture medium (Gibco) supplemented with 10% FBS (Gibco), 100 units/ml of penicillin (Gibco), 100 μg/ml of streptomycin (Gibco) and incubated at 37°C in 5% CO_2_ humidified incubator. U‐87 MG cells were maintained in EMEM cell culture medium supplemented with 1% Non‐Essential Amino Acids (NEAA) (Gibco), 10 mM HEPES (Gibco), 10% FBS (Gibco), 100 units/ml of penicillin (Gibco), 100 μg/ml of streptomycin (Gibco) and incubated at 37°C in 5% CO2 humidified incubator. J3T‐BG, SDT3G and G06A cells were grown in RPMI 1640 (Gibco) supplemented with 10% FBS (Gibco), 100 units/ml of penicillin (Gibco), 100 μg/ml of streptomycin (Gibco) and incubated at 37°C in 5% CO_2_ humidified incubator.

### 2.2 Temozolomide

Temozolomide was obtained from Sigma‐Aldrich (Tecris, MN, USA). The stock solution was prepared by dissolving in DMSO to get a 50 mM concentration, the aliquots were stored at −20°C and used within 1 month. For the proliferation assay 50 mM, temozolomide was diluted in sterile PBS and for the clonogenic assay in medium of the treated cell line to reach the indicated doses of temozolomide. DMSO diluted in the same amount of PBS or medium served as solvent control for temozolomide treatment.

### 2.3 Irradiation

Irradiation was performed with 6 MV linear accelerator (Clinac iX, Varian, Palo Alto, USA), a field size of 30 × 30 cm and source‐surface distance (SSD) of 100 cm and a dose‐rate of 600 monitor units per minute. Adequate dose build‐up and optimal homogeneity of the dose distribution over the irradiation field was ensured by appropriate layers of plexiglass. Dose is routinely checked and calibrated by a board‐certified medical physicist.

Human glial tumour cells as well as J3T‐BG and G06A were irradiated at doses of 4 and 8 Gy, SDT3G with doses of 2, 4 and 8 Gy.

### 2.4 Proliferation assay

Note that 1000 cells for J3T‐BG, 2000 cells for A172, U‐87 MG and SDT3G and 4000 cells per well for G06A were seeded into 96‐well plates the day before treatment to allow for attachment of cells. For initial dose finding of chemotherapeutic agent, cells were treated with 100, 200 and 400 μM of temozolomide. U‐87 MG was treated with 10 and 20 μM of temozolomide. For initial dose finding of irradiation, cells were treated with 4 and 8 Gy. For the combination studies, temozolomide was added to cells 24 h prior to irradiation, cells were treated with 4 and 8 Gy and the cell proliferation was measured 48 h after irradiation. The cells were incubated with temozolomide for the duration of the experiment (no medium exchange after irradiation). Time point zero was defined as the time directly after irradiation. The proliferation assay was performed using a cell counting kit CCK‐8 according to the manufacturer's protocol (Dojindo Laboratories). CCK‐8 reagent is a ready for use solution, which allows determination of the number of viable cells in proliferation assays. Note that 10 μl of the CCK‐8 solution was given to the corresponding wells and absorbance was measured 3 h later at 450 and 600 nm using microplate reader Epoche 2 (BioTek, Winooski, Vermont, USA). The experiments were performed at least three times (with each experiment including three technical replicates).

### 2.5 Clonogenic cell survival assay

**FIGURE 1:**
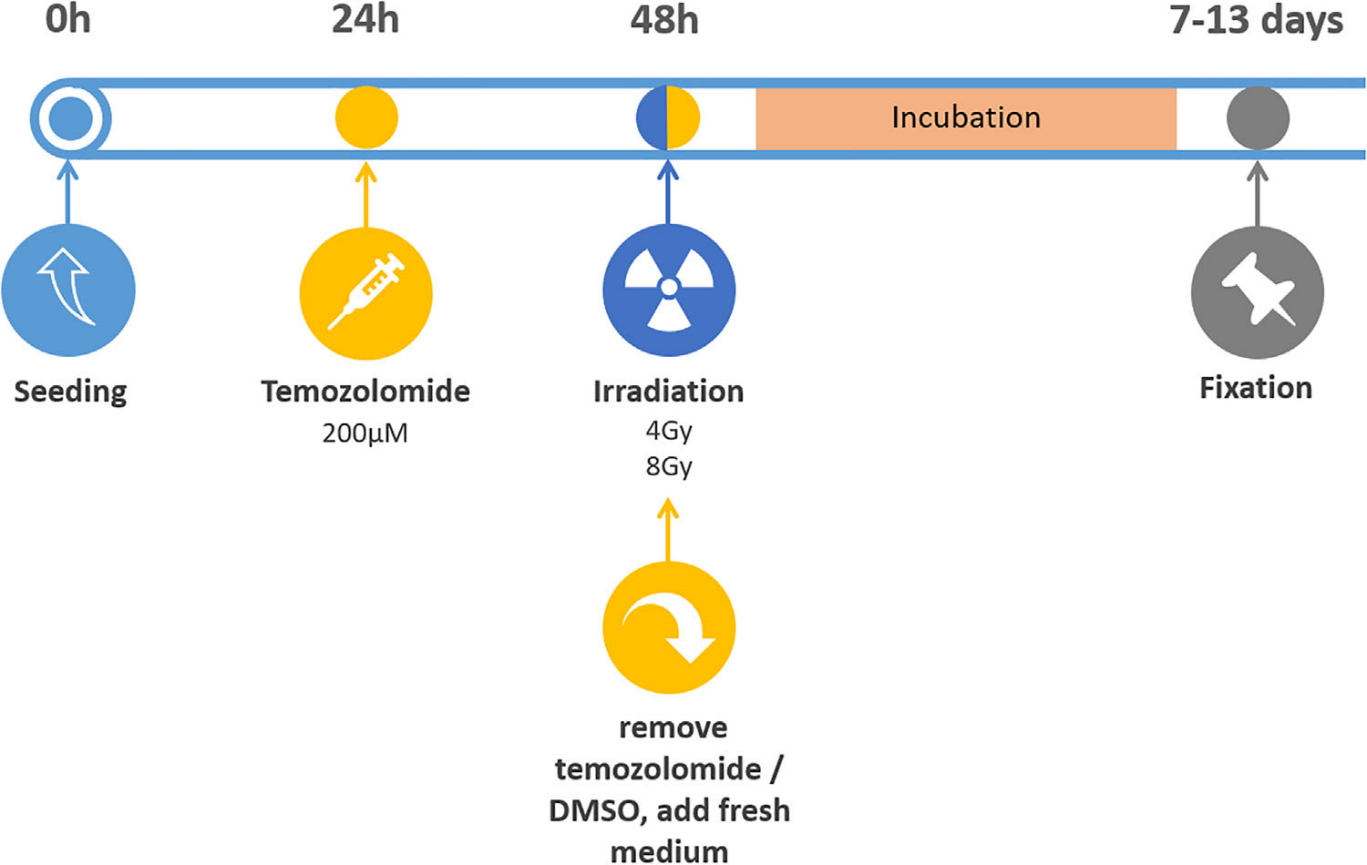
Timeline of the experiment setting for clonogenic assay in canine cell lines and U‐87 MG with temozolomide 24 h prior to irradiation

Clonogenic cell survival was determined in the cells treated with radiotherapy alone or combined with temozolomide. Cells were seeded into 10 cm petri‐dishes 24 h before temozolomide administration. The cell number and temozolomide concentration to reach optimal (separate single clones) number of clones was optimized for every cell line and every condition beforehand: J3T‐BG 200 cells (0 Gy), 400 cells (4 Gy) and 4000 cells (8 Gy); SDT3G 200 cells (0 Gy), 1000 cells (4 Gy) and 4000 cells (8 Gy); and G06A 500 cells (0 Gy), 2000 cells (4 Gy) and 4000 cells (8 Gy). For U‐87 MG, we used different cell numbers in plates treated with DMSO and temozolomide: 200 cells (0 Gy, DMSO), 400 cells (0 Gy, TMZ), 800 cells (4 Gy, DMSO), 1600 cells (4 Gy, TMZ), 4000 cells (8 Gy, DMSO) and 8000 cells (8 Gy, TMZ). Twenty‐four hours after treatment with temozolomide, cells were irradiated with 4 and 8 Gy (Figure 1). After irradiation, the medium containing temozolomide as well as the medium in control dishes was removed and replaced by fresh medium. Based on the results of the proliferation assay, we chose a dose of 200 μM of temozolomide for the canine cell lines. Additionally, reversed order of the treatment, namely first irradiation and then incubation with temozolomide was performed in J3T‐BG cells (Figure S1). Temozolomide was administered immediately after irradiation using the same treatment doses as described above. Temozolomide and DMSO control were removed and replaced by fresh medium after 24 h.

In U‐87 MG cells treated with 200 μM, no clones were formed which is why we reduced the TMZ dose to 10 μM. We could not obtain accountable number of clones for A172 human canine cell line; therefore, we used only U‐87 MG cell line as a human cell line comparison.

After colony formation (9–10 days (J3T‐BG), 11 days (SDT3G), 7 days (G06A), 13 days (U‐87 MG) after seeding), colonies were fixed (methanol/acetic acid; 3:1) and stained with crystal violet (1%, Sigma). Colonies containing >50 cells were counted manually using a colony counter. Cell‐surviving fraction was calculated by dividing the number of obtained colonies after treatment by the number of seeded cells and correcting for plating efficiency of control cells using the formula: SF (Surviving fraction) = number of colonies formed after treatment/number of cells seeded × PE (plating efficiency).

### 2.6 Genomic DNA isolation and *MGMT* methylation analysis

Cell lysates of the canine cell lines (J3T‐BG, G06A and SDT3G) were generated with the PureLink Genomic DNA Mini Kit (Thermo Fischer Scientific) according to the manufacturer's protocol. Total DNA (200–500 ng) was treated with sodium bisulfite using the EZ DNA Methylation‐Lightning Kit (Zymo Research Europe, Freiberg, Germany) according to the manufacturer's protocol. Target enrichment for the *MGMT* promoter, exon 1 and enhancer was performed by a two‐step PCR protocol as previously described (Morandi et al., 2020). In brief, locus‐specific amplicon libraries were generated with tagged primers with a first round of PCR amplification followed by a second fast round (8 cycles) for barcoding using the Nextera Index Kit (Illumina). The regions of interest included the following coordinates taking into account the reference genome canFam3 (UCSC, Genome Browser): chr28:38203631‐38203869 (promoter); chr28: 38204614–38204813 (exon 1); chr28:37634643‐37634834 (enhancer).

MethPrimer (http://www.urogene.org/cgi‐bin/methprimer/methprimer.cgi) designing was applied to identify CpGs and the primers of choice. The sequencing was conducted on MiSeq sequencer (Illumina) according to the manufacturer's protocol. Each next‐generation sequencing (NGS) experiment was designed to allocate at least 1000 reads/region in order to have a depth of coverage of at least 1000×.

FASTQ files were processed in a Galaxy Project environment by the tool Filter by Quality for the quality control (>Q 30) and Filter FASTQ reads for read lengths (>80 bp). FASTQ files were then mapped by BWAmeth, generating bam files which were in turn processed by MethylDackel using CanFam3.1 as reference genome. This tool created a file for each case, assigning the exact methylation level for each investigated CpG position (Afgan et al., 2018).

Quantitative DNA methylation data were analyzed using methylation plotter (https://gattaca.imppc.org/methylation_plotter/) (Mallona et al., 2014).

### 2.7 Western blot

For immunoblotting analysis, cells were washed with cold PBS, harvested and centrifuged 5 min at 1300 × *g* in a cold microfuge. Supernatant was removed and the pellets were resuspended in 50 μl of RIPA lysis buffer (Sigma‐Aldrich) containing protease inhibitor cocktail (Sigma‐Aldrich). Cells were lysed for 15 min on ice with occasional vortexing. The cell debris was pelleted at 13,000 × *g* in a cold microfuge for 10 min and supernatant were stored at −20 until analysis.

The samples were separated on a 4%–15% gradient gel (Bio‐Rad) and blotted on PVDF membrane using a transfer apparatus according to manufacturer's protocol (Bio‐Rad). After blocking with 5% non‐fat milk in TBST (Bio‐Rad), membranes were probed with MGMT antibody (1:500, Milipore, Billerica, MA, USA); and secondary anti‐mouse IgG, HRP‐linked antibody (#7076, 1:1000, Cell Signaling Technology). The protein was visualized using Pierce ECL Western Blotting Substrate (Thermo Fisher Scientific) and exposed to x‐ray film. After washing and blocking with 5% non‐fat milk in TBST (Bio‐Rad), membranes were probed with β‐actin antibody (#8226, 1:1000, Abcam) and secondary anti‐mouse IgG, HRP‐linked antibody (#7076, 1:000, Cell Signaling Technology) to confirm successful cell lysis. As a positive control, recombinant human MGMT protein was used (ab136378; Abcam). Moreover, in order to ascertain that the MGMT antibody and the experimental setting were able to detect human and canine MGMT protein correctly, MGMT was analyzed in other canine and human tumour cell lines lysates (canine osteosarcoma cell line OSA17 and human lung carcinoma A549).

### 2.8 Statistical analysis

Statistical analyses were performed using GraphPad Prism Version 8 (San Diego, CA, USA). The analysis of the data obtained from proliferation assays was done with one‐way ANOVA and Tukey's post‐hoc test was used for comparison of the four groups to each other. Unpaired *t*‐test was used to compare two groups to each other analyzed by the clonogenic cell survival assay and proliferation assay. The *MGMT* methylation data were analyzed using methylation plotter (https://gattaca.imppc.org/methylation_plotter/), and statistical significance was calculated using Kruskal–Wallis test (Mallona et al., 2014). *p*‐Values below 0.05 were considered statistically significant and denoted with a star (*), two stars (**) were used for *p*‐values below 0.005 and three stars (***) were used for *p*‐values falling below 0.001.

## 3 RESULTS

### 3.1 Antiproliferative effects of different temozolomide and radiotherapy treatment dose in human and canine glial tumour cell lines

We tested the effect of temozolomide and radiotherapy in the panel of human and canine glial tumour cell lines using proliferation assay in order to determine the optimal concentration for the combinatorial experiments.

**FIGURE 2:**
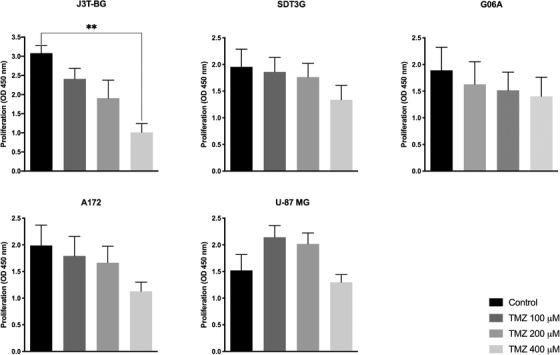
Effect of temozolomide treatment on cell proliferation in canine (J3T‐BG, SDT3G, G06A) and human (A172, U‐87 MG) glioma cell lines. Cell viability was measured at time point time point 48 h after treatment. Mean ± SEM at least of three experiments performed independently is shown

Temozolomide had concentration‐dependent inhibitory effect on cell proliferation in all three cell lines tested (Figure 2). The cell lines, however, were unequally sensitive towards temozolomide: At doses of 100, 200 and 400 μM cell, survival in J3T‐BG was reduced to 77, 60 and 33%; in SDT3G to 97, 93 and 68%; and in G06A to 85, 80 and 73%, respectively. In human glial tumour cells, at doses of 100, 200 and 400 μM, A172 was inhibited to 92, 86 and 60%; in U‐87 MG 100 and 200 μM temozolomide did not inhibit cell proliferation, a reduction to 91% in cells treated with 400 μM was reported.

**FIGURE 3:**
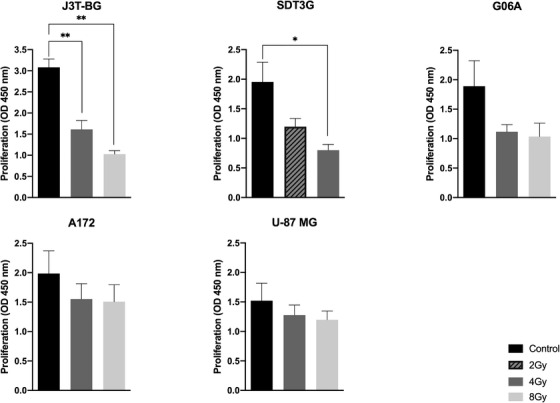
Effect of irradiation on cell proliferation in canine (J3T‐BG, SDT3G, G06A) and human (A172, U‐87 MG) glioma cell lines. Cell viability was measured at time point time point 48 h after treatment. Mean ± SEM of at least three experiments performed independently is shown

Note that 4 and 8 Gy reduced the proliferation of J3T‐BG cells to 52% and 33% and G06A to 63% and 56%, respectively (Figure 3). At the dose of 4 Gy, SDT3G was inhibited to 43%. Due to the higher radiosensitivity of this cell line compared to the other canine cell lines, we tested 2 Gy instead of 8 Gy (inhibition down to 23%) which diminished the proliferation to 63%. Human glial tumour cells were tested with doses of 4 and 8 Gy. Cell survival of A172 was decreased to 80% and 77%; and U‐87 MG to 88% and 83%, respectively (Figure 3).

Based on our findings, we selected the same temozolomide doses for all cell lines to be used in combination experiments. The effect of combination in SDT3G was tested with radiation doses of 2 and 4 Gy. The remaining canine and human glial cell lines were tested with doses of 4 and 8 Gy.

### 3.2 Combination of irradiation and temozolomide administration shows no significant antiproliferative effect compared to radiation or temozolomide alone

**FIGURE 4:**
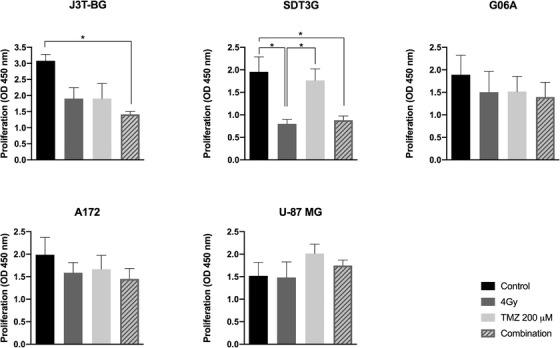
Combination of temozolomide 200 μM and irradiation at the dose of 4 Gy does not result in enhanced cancer cell growth inhibition in canine (J3T‐BG, SDT3G, G06A) and human (A172, U‐87 MG) glial tumour cell lines. Cells pre‐treated with temozolomide were incubated for 24 h before irradiation. Cell viability was measured at time point time point 48 h after irradiation. Mean ± SEM of at least three experiments performed independently is shown

Combined treatment significantly reduced the proliferation in comparison to control cells in J3T‐BG and SDT3G; however, it did not significantly reduce proliferation in comparison to single treatments with 200 μM TMZ and 4 Gy (Figure 4). We did not observe additive effects of combined treatment in G06A, U‐87 MG and A172 cell lines (Figure 4). Additionally, we performed the assay with other TMZ and radiation dose combinations (Figures S2–S4), as above, we only observed a significant difference between combined treatment and control in J3T‐BG and SDT3G cell lines. In U‐87 MG, we also tested radiation dose of 4 or 8 Gy combined with 10 or 20 μM TMZ (Figure S5). There was no significant reduction of proliferation in combined treatment compared to single treatment or control.

### 3.3 Combined treatment significantly inhibits canine glial cell clonogenicity in comparison to radiation alone

**FIGURE 5:**
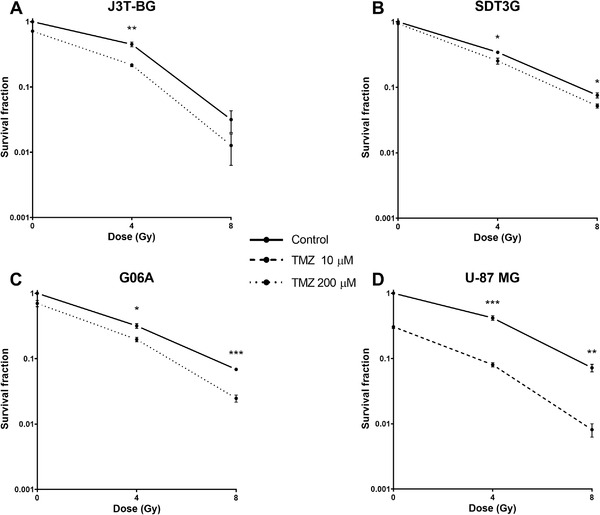
Clonogenic survival of canine glioma cell lines (a) J3T‐BG, (b) SDT3G, (c) G06A and (d) human U‐87 MG after irradiation with 4 or 8 Gy ±200 μM temozolomide (TMZ) for canine cell lines or ±10 μM temozolomide (TMZ) for U‐87 MG, respectively. Surviving fraction (SF) calculated using mean plating efficiency (PE) of untreated cells as denominator to illustrate independent cytotoxic effects of TMZ. Mean ± SEM of at least three independent experiments performed independently is shown

Survival curves for the three canine glioma cell lines and human U‐87 MG treated with radiation, TMZ alone, and concomitantly with 200 μM TMZ and 10 μM TMZ, respectively, are presented in Figure 5. We used lower TMZ dose for U‐87 MG because we obtained no clones with 200 μM TMZ. Canine cell lines J3T‐BG, SDT3G and G06A showed a significant difference following combined treatment with TMZ at dose of 200 μM and irradiation with 4 Gy, compared to radiation alone. The difference was also significant in combination with 8 Gy in SDT3G and G06A cell lines. Combination of 10 μM TMZ and 4 and 8 Gy led to a significant decrease in cell survival in U‐87 MG. Interestingly, we observed no significant differences in clonogenic cell survival of J3T‐BG cells incubated with TMZ before or after irradiation, suggesting that the treatment order does not play a role in the radiosensitization outcome in this cell line (Figure S6). Combination of 10 μM TMZ and 4 and 8 Gy led to a significant decrease in cell survival in U‐87 MG. In summary, all three canine cell lines as well as U‐87 MG benefit from the combined treatment of irradiation and TMZ, resulting in a decrease in the number of surviving cells.

### 3.4 *MGMT* promoter, enhancer and exon 1 methylation levels in canine glial tumour cell lines

**FIGURE 6:**
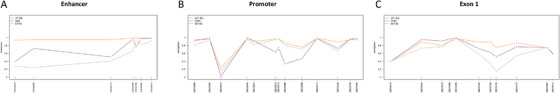
Methylation plots of *MGMT* enhancer (a), promoter (b) and exon 1 (c) of the three canine glioma cell lines. Three samples of each cell line were analyzed. Each line represents the methylation mean for each position for every cell line (three samples analyzed per cell line). Asterisks indicate a statistical significance as calculated by the Kruskal–Wallis test

To study the role of MGMT in canine glial tumour cell lines in response to treatment, we measured the levels of *MGMT* methylation in the three canine cell lines. We observed variations in methylation levels in *MGMT* enhancer between the three cell lines, especially J3T‐BG showing low levels depending on the location of methylation (Figure 6a). When methylation of *MGMT* promoter was analyzed, with exception to one location, all three canine cell lines were highly methylated, although G06A showed variation from the other two cell lines (Figure 6b). Exon 1 of *MGMT* also had high levels of methylation with J3T‐BG showing the lowest levels (Figure 6c). We have also analyzed the MGMT protein levels in these cell lines using antibodies recognizing human MGMT. As shown previously, A172 and U‐87 MG did not show a detectable level of MGMT protein (Perazzoli et al., 2015; Ryu et al., 2012). Similarly, none of the three tested canine glial tumour cell lines (J3T‐BG, SDT3G and G06A) had detectable MGMT levels in comparison to recombinant MGMT control and A549 cell lysate (Figure S7). As a positive control, we used recombinant human MGMT protein and protein lysates of human pulmonary adenocarcinoma cell line A549 which has been previously described with high level of MGMT protein (Wu et al., 2001).

## 4 DISCUSSION

In humans, glioblastoma therapy includes maximal safe resection, followed by radiation therapy and temozolomide chemotherapy (Ali et al., 2020; Mann et al., 2017; Nam & De Groot, 2017). The addition of temozolomide has increased median survival of human brain tumour patients to 14.6 months compared to 12.1 months with radiotherapy alone (Stupp et al., 2009). Already in human glioblastoma cell lines in vitro, temozolomide has been found to exhibit additive toxicity in combination with ionizing radiation (Chalmers et al., 2009). In dogs, a surgical approach is often not possible at all, and chemotherapy has rarely been used. In the veterinary clinical setting, no survival advantage could be found in dogs irradiated for glial tumors in combination with temozolomide (at low doses of 65 mg/m^2^ daily in a five‐day cycle) (Dolera et al., 2018).

### 4.1 Effect of temozolomide in combination with radiation on canine glioma cell clonogenic cell survival

In our study, we found an additive cytotoxic effect of temozolomide and irradiation in all three canine glioma cell lines as measured by clonogenic cell survival assay. A schedule‐dependent (temozolomide added before or after irradiation) effect of combined treatment as described in human cell lines was not detected in J3T‐BG cell line. (Chalmers et al., 2009) We chose the 24 h pre‐incubation with temozolomide in clonogenic assay experiments temozolomide as previous studies with human cancer cell lines showed this incubation time was optimal time to see the inhibitory effect (Ma et al., 2011; Mirabdaly et al., 2020). Moreover, we observed that when temozolomide was not removed from the cells after 24 h incubation (like in proliferation assay), no clones were formed in the temozolomide‐treated plates. It could be attributed to the fact that proliferation assay measures only short‐time growth potential (up to 48 h after irradiation) of the cells, whereas clonogenic cell survival assay reflects long‐term growth and clone survival capacity (up to 13 days depending on the cell line) and therefore longer exposure to temozolomide is more cytotoxic. Interestingly, contrary to the proliferation assay, in the clonogenic assay of U‐87 MG, we determined a high sensitivity towards temozolomide. Similar results and the need for dose reduction in the clonogenic survival assay in human glioma cell lines have been described before (Baer et al., 1993; Hermisson et al., 2006; Li et al., 2018; Montaldi et al., 2015; Ryu et al., 2012).

### 4.2 Effect of temozolomide alone and in combination with radiation on canine glioma cell proliferation

We observed an inhibition of cell proliferation of J3T‐BG comparable to previously reported results (Boudreau et al., 2017). In G06A and SDT‐3G, chemosensitivity and a visible reduction of cell viability in cells treated with lomustin n‐(2‐chloroethyl)‐n‐cyclohexyl‐n‐nitrosourea (CCNU) have been described before, but there was no report about specific temozolomide sensitivity in these two cell lines (Boudreau et al., 2017). Radiosensitivity has only been tested in J3T CD133 positive cancer stem cells with our results being in line with the reported cell viability of adherent cells (Pang et al., 2017).

For proliferation assay, we chose a 48‐h time point as described in previous studies in human glial tumour cell lines treated with temozolomide (Borhani et al., 2017). However, the changes in proliferation might be observed at later time points, not analyzed here.

In U‐87 MG treated with temozolomide with or without irradiation, clonogenic assays, cell proliferation and MTT cell viability assays yielded similar findings as prior described. In spite of the variation in experimental settings, behaviour of the human glioma cell line U‐87 MG was comparable to those reported before with a relatively high resistance towards irradiation and a varying sensitivity towards temozolomide (Baer et al., 1993; Borhani et al., 2017; Hermisson et al., 2006; Montaldi et al., 2015; Ryu et al., 2012). Our results of proliferation assay in A172 are not in line with previous reports, describing relatively high sensitivity towards temozolomide, while in our experiments, the cell line seemed less temozolomide‐responsive (Borhani et al., 2017; Lee, 2016).

### 4.3 Role of MGMT in temozolomide and radiation response in glioma cells

One of the most important causes for treatment failure is drug resistance of tumour cells (Stupp et al., 2009). Resistance to temozolomide has previously been related to MGMT expression levels in vivo and in vitro (Hegi et al., 2005, 2004; Hermisson et al., 2006; Perazzoli et al., 2015). *MGMT* promoter methylation (silencing of the promoter), however, leads to incomplete DNA‐repair capacity and chemo‐radiation treatment is more damaging (Sharma et al., 2010). Concerning temozolomide, low MGMT protein expression has been described being even more predictive than *MGMT* promoter methylation itself (Van Nifterik et al., 2010). Previous studies showed increased double strand DNA damage in MGMT‐negative glioblastoma cell lines treated with RT combined with temozolomide compared to single treatment (Chakravarti et al., 2006).

MGMT‐coding regions in dogs are 78% similar to human MGMT‐coding regions with about 62% amino acid identity and an importance in resistance to cytotoxic drugs similar to the human MGMT was suspected. (Zaboikin et al., 2004) In canine lymphoma cells, however, the expression of MGMT mRNA was found to be not associated with chemosensitivity (Tomiyasu et al., 2010). One study tested canine lymphoma cells for *MGMT* methylation status and could not report a significant relation to MGMT activity (Kambayashi et al., 2015). On the other hand, a correlation between MGMT and resistance to the alkylating agent CCNU as well as an increased sensitivity to this drug in canine lymphoma cells cultured with a MGMT inhibitor has been reported (Kambayashi et al., 2015).

*MGMT* mRNA expression was measured in G06A, SDT‐3 and J3T‐BG; the latter two cell lines showed high *MGMT* mRNA expression level (Chakkath et al., 2015). Canine lymphoma cells and canine hepatocytes were tested for *MGMT* mRNA and enzyme activity, and a positive correlation between *MGMT* mRNA expression and enzyme activity was found (Chakkath et al., 2015). In our analysis of *MGMT* promoter methylation in three canine cell lines, we detected very high methylation levels (with exception to one location), interestingly, the methylation of enhancer and Exon 1 has shown higher variability between cell lines. The high methylation levels of *MGMT* promoter can explain lack of MGMT protein expression in these cell lines. On the other hand, the antibodies used for the detection of MGMT were generated against human protein; therefore, we cannot exclude that the lack of signal could be due to low or no cross‐reactivity with canine MGMT.

### 4.4 Limitations

Some limitations of this study should be noted. First, we only used three different canine cell lines for our experiments. All of them as well as the human glioma cell lines had no detectable level of MGMT protein. Therefore, we were unable to compare cells with different MGMT protein level in chemo‐ and radiosensitivity. Second, the alkylating agent temozolomide has been used as a therapeutic option for different tumour types in dogs. In vivo, myelosuppression as a systemic side effect of oral temozolomide is dose‐limiting (Newlands et al., 1992). A tolerated dose of 60–100 mg/m2 oral temozolomide once daily for 5 days on a 28‐day cycle has been described before with occurring side effects concerning haematology and gastrointestinal tract. The maximally tolerated dose for temozolomide for dogs has just recently been established at 150 mg/m^2^ daily in a 5‐day cycle and should serve as a future reference dose (Marconato et al., 2020). In our experiments, we used concentrations of temozolomide between 100 and 400 μM. Concentrations of temozolomide over 100 μM are higher than plasma levels achieved during oral temozolomide chemotherapy described in human tumour patients and therefore only used in vitro (Knizhnik et al., 2013; Ostermann et al., 2004). We do not know if a similar additive cytotoxic effect could have been observed at lower temozolomide concentration levels. Recently, an experimental intratumoral temozolomide therapy in dogs with spontaneous gliomas was described with the aim to achieve higher intracranial TMZ levels while avoiding systemic side effects (Hicks et al., 2017; Hicks et al., 2019). Therefore, effective concentrations of temozolomide similar to our in vivo study might be achievable.

In conclusion, in the tested canine glial tumour cell lines, temozolomide was additive to the effects of irradiation in clonogenic cell survival assay and *MGMT* promoter was highly methylated. It will be of future interest if a methylated promotor of MGMT is the reason for this undetectable protein level in the three canine cell lines or if the expression of MGMT is regulated by mechanisms independent of *MGMT* promoter methylation (Blough et al., 2011; Perazzoli et al., 2015; York et al., 2018).

## CONFLICT OF INTEREST

The authors declare no conflict of interest.

## AUTHOR CONTRIBUTIONS

*Investigation*: Daniel Fuchs. *Investigation, methodology and writing‐review & editing*: Luca Morandi. *Formal analysis and methodology*: Caterina Tonon. *Conceptualization, supervision, writing‐original draft and writing‐review & editing*: Carla Rohrer Bley.

## Supporting information

Figure S1

Figure S2

Figure S3

Figure S4

Figure S5

Figure S6

Figure S7

## Data Availability

The data that support the findings of this study are available on request from the corresponding author.
