# Supplementary figures and images for: Temozolomide is additive with cytotoxic effect of irradiation in canine glioma cell lines
Source: Vet Med Sci. 2021 Sep 3;7(6):2124–34. doi: 10.1002/vms3.620 (PMC8604143; doi:10.1002/vms3.620)

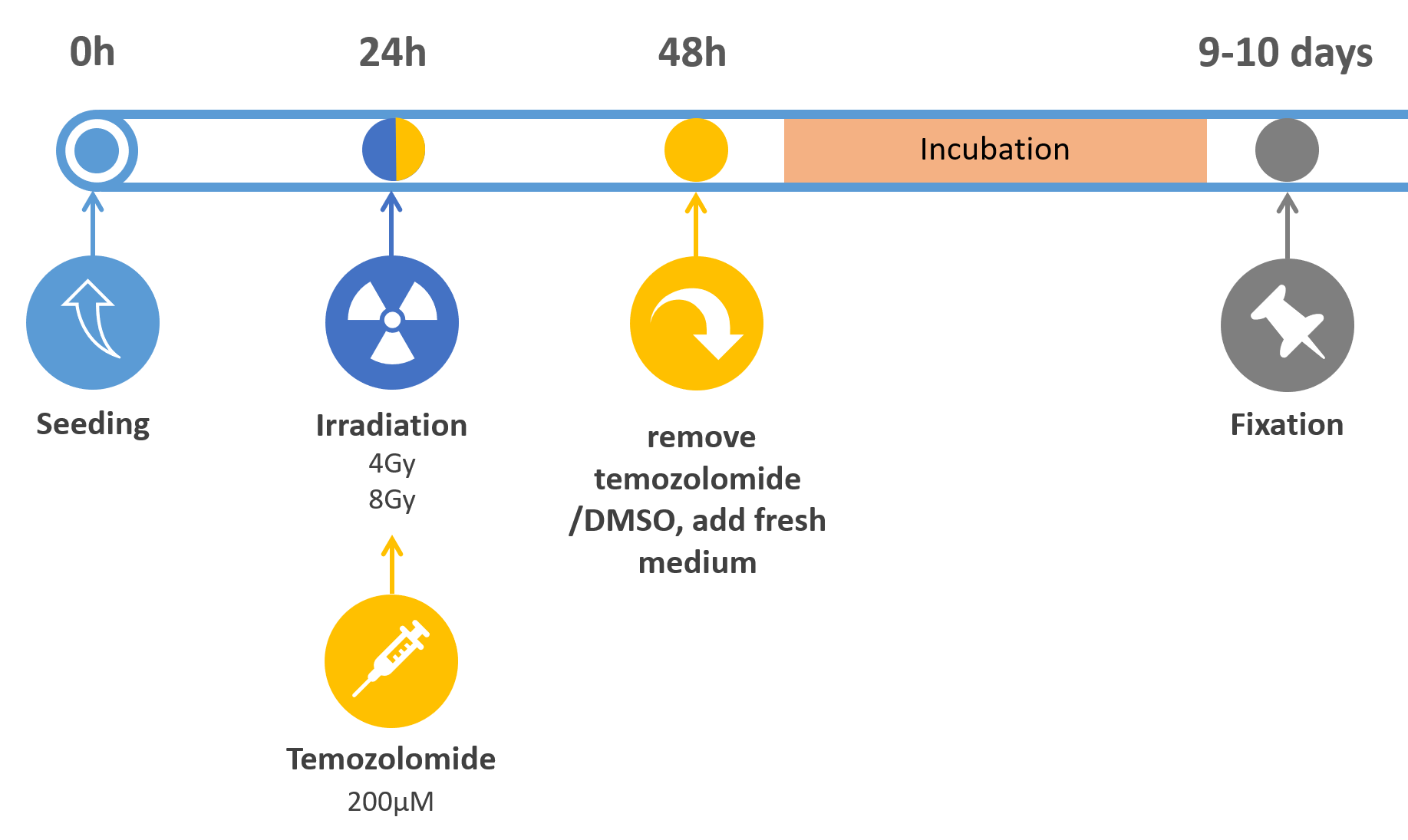

Supplement: Supplementary file 1 — Figure S1 [file VMS3-7-2124-s007.tiff]

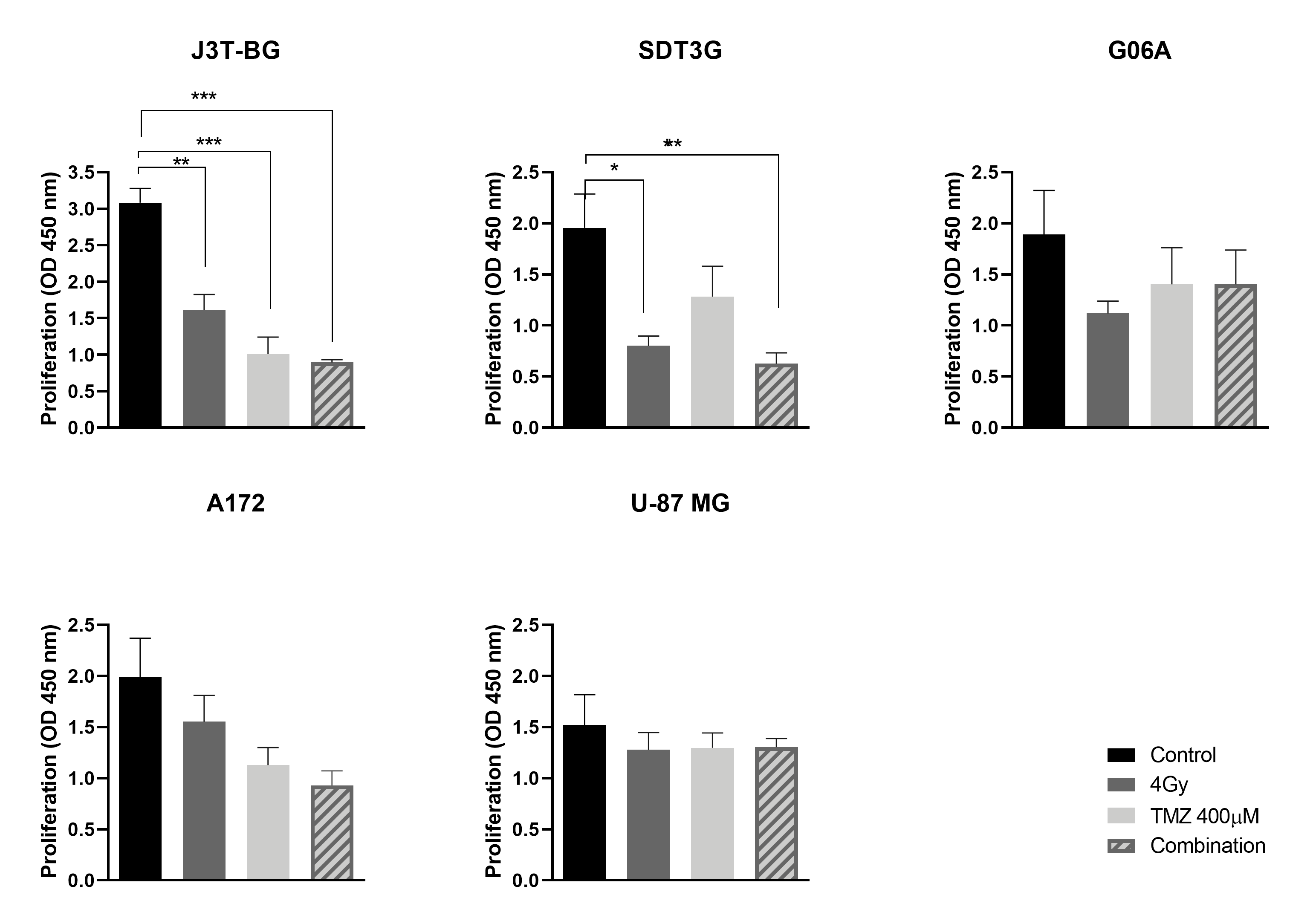

Supplement: Supplementary file 2 — Figure S2 [file VMS3-7-2124-s005.tif]

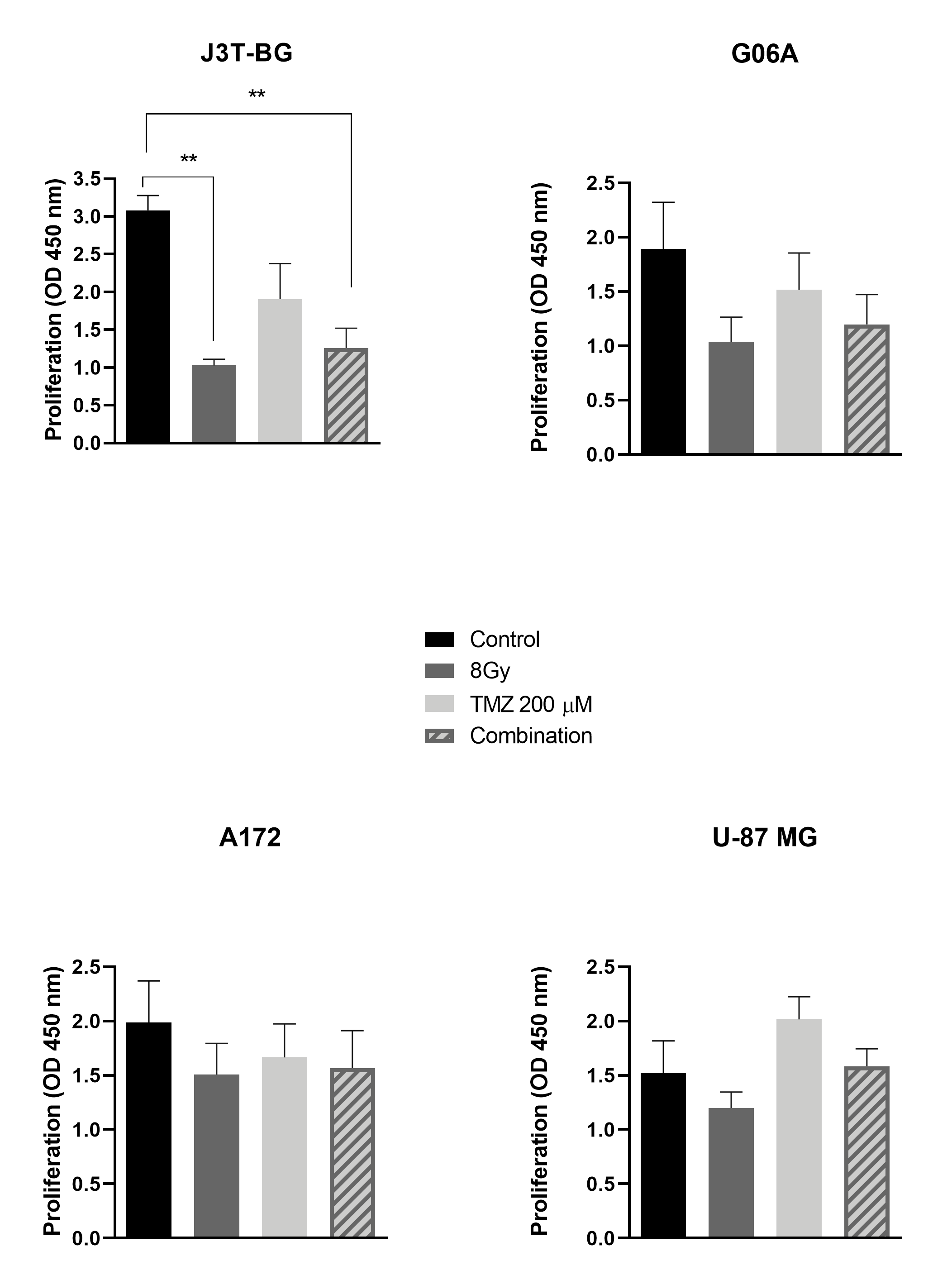

Supplement: Supplementary file 3 — Figure S3 [file VMS3-7-2124-s004.tif]

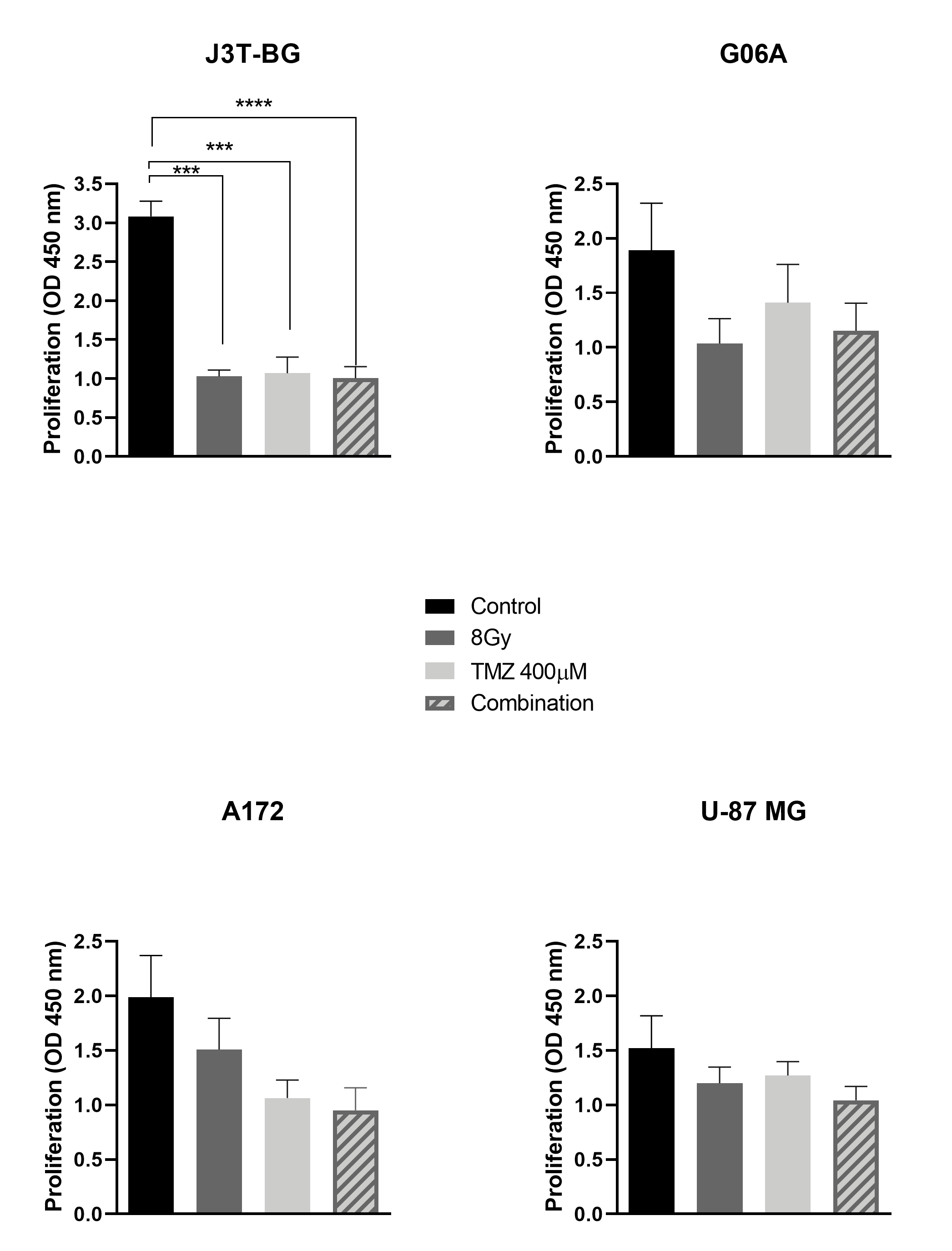

Supplement: Supplementary file 4 — Figure S4 [file VMS3-7-2124-s006.tif]

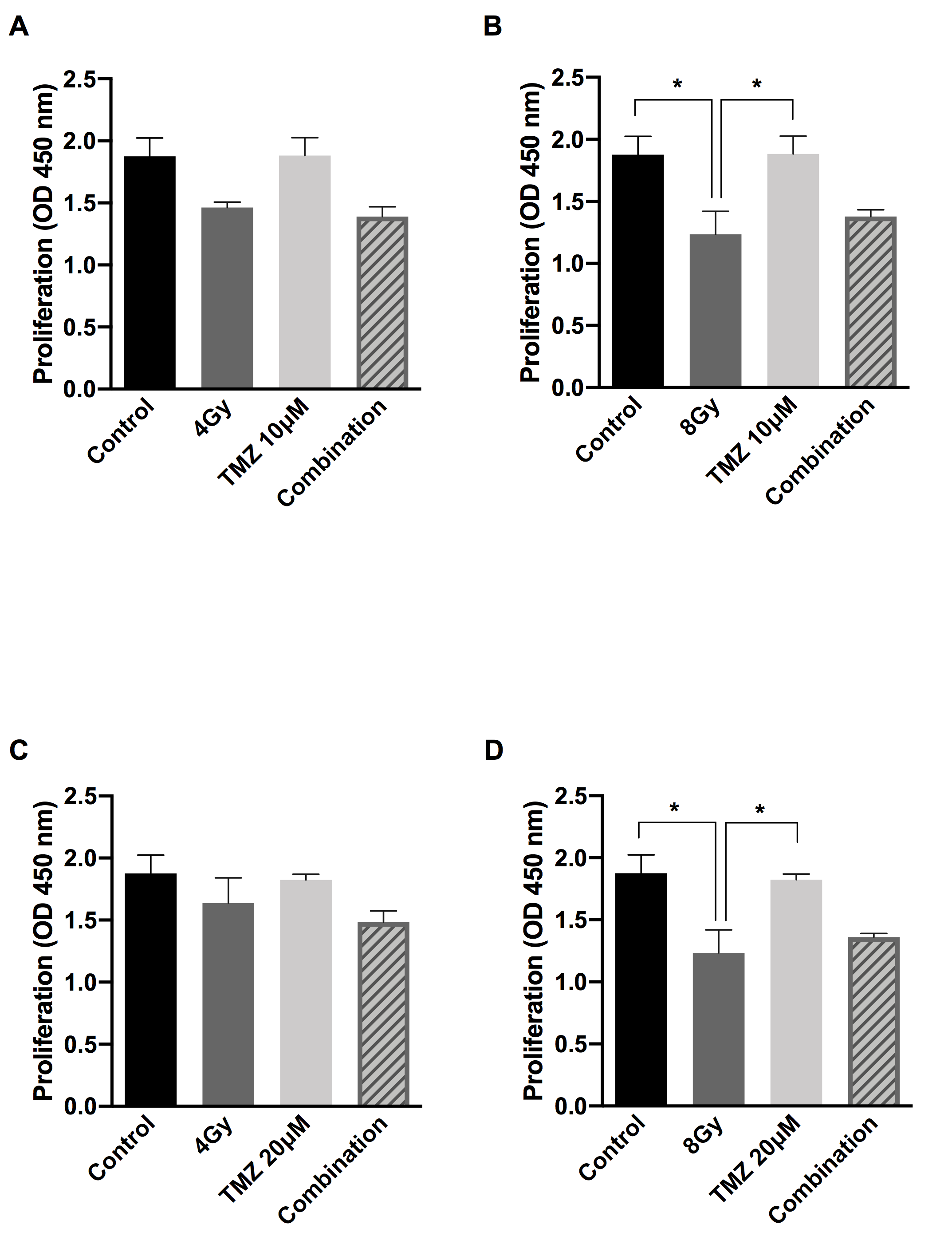

Supplement: Supplementary file 5 — Figure S5 [file VMS3-7-2124-s003.tiff]

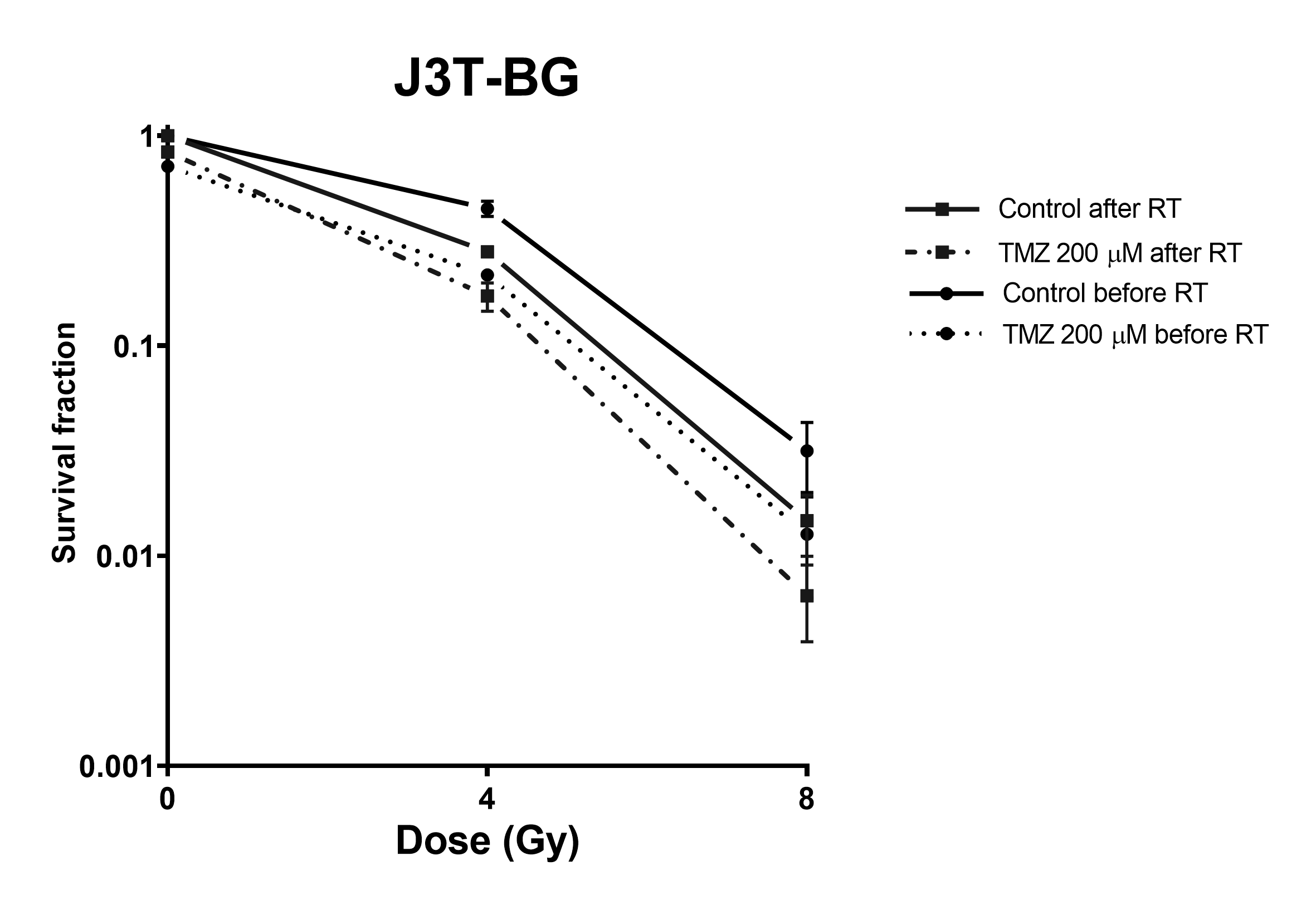

Supplement: Supplementary file 6 — Figure S6 [file VMS3-7-2124-s001.tif]

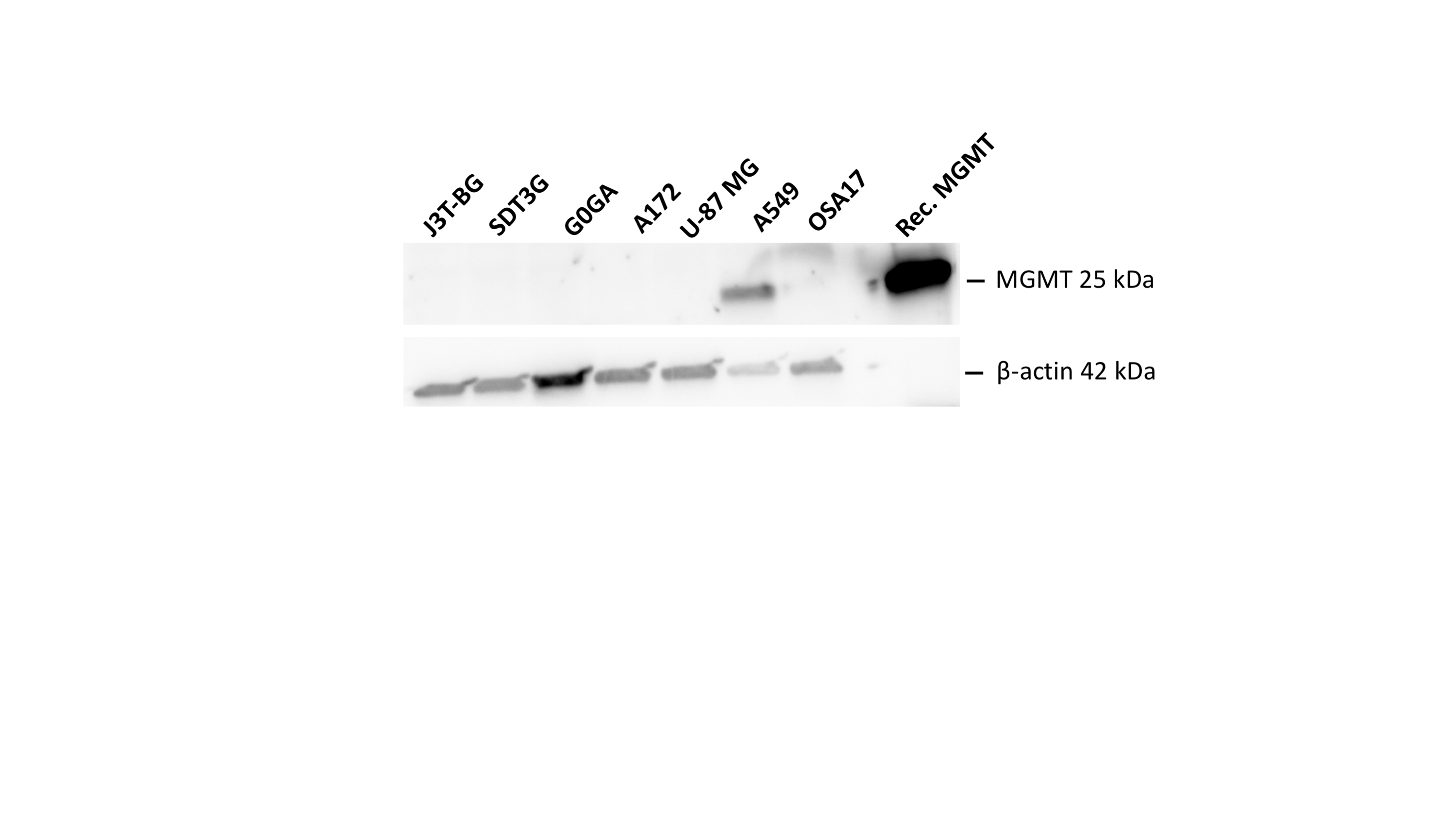

Supplement: Supplementary file 7 — Figure S7 [file VMS3-7-2124-s002.tiff]
